# Trans-sylvian hematoma evacuation combined with lamina terminalis ventriculostomy for hypertensive basal ganglia hemorrhage: a retrospective cohort study

**DOI:** 10.3389/fsurg.2026.1874525

**Published:** 2026-08-04

**Authors:** Liu Huang, Qiurong Yang, Chengping Wu, Runsan Hu, Ying Tang

**Affiliations:** 1 Department of Neurosurgery, Xiangdong Hospital Affiliated to Hunan Normal University, Zhuzhou City, Hunan Province, China; 2 Department of Gastroenterology, Xiangdong Hospital Affiliated to Hunan Normal University, Zhuzhou City, Hunan Province, China

**Keywords:** cerebral hemodynamics, hypertensive basal ganglia hemorrhage, intraventricular extension, lamina terminalis ventriculostomy, trans-sylvian approach

## Abstract

**Background:**

Hypertensive basal ganglia hemorrhage (HBGH) with intraventricular extension is frequently associated with cerebrospinal fluid (CSF) circulation disturbance, postoperative complications, and poor neurological outcomes. We evaluated a single-stage surgical strategy combining trans-sylvian hematoma evacuation with lamina terminalis ventriculostomy (LTV) in patients with HBGH and ventricular extension.

**Methods:**

This retrospective cohort study included 70 patients with HBGH (hematoma volume ≥30 mL) and intraventricular extension. Patients who underwent isolated trans-sylvian hematoma evacuation between 2020 and 2021 served as the control group (*n* = 35), whereas patients treated with trans-sylvian evacuation plus LTV between 2023 and 2024 comprised the combined group (*n* = 35). Propensity score matching (1:1.5 nearest-neighbor matching, caliper = 0.2) was performed using available baseline variables. Cerebral hemodynamics, ADL-based functional recovery, postoperative complications, and EORTC QLQ-C30 scores were analyzed.

**Results:**

After matching, 20 control patients and 30 combined-procedure patients were included. The combined group showed significantly higher postoperative mean flow velocity and lower pulsatility index at postoperative days 7 and 14 (all *P* < 0.001). Functional recovery assessed by ADL grades I–II was significantly better in the combined group (76.67% vs. 35.00%, *P* = 0.003). The overall incidence of postoperative complications was significantly lower in the combined group (6.67% vs. 55.00%, *P* < 0.001). Although significant differences in EORTC QLQ-C30 scores were observed during follow-up, these findings did not consistently favor the combined procedure and should be interpreted cautiously.

**Conclusion:**

Trans-sylvian hematoma evacuation combined with LTV was associated with improved cerebral hemodynamics, better ADL-based functional recovery, and fewer postoperative complications in selected patients with HBGH and ventricular extension. Because several severity-related variables and shunt-dependency outcomes were unavailable, prospective multicenter studies are required to confirm its long-term clinical benefits.

## Background

Hypertensive basal ganglia hemorrhage (HBGH) is an acute pathological process characterized by cerebrovascular rupture due to hypertension, leading to hematoma formation in the basal ganglia (1). As a critical region regulating coordinating motor, emotional and cognitive functions, basal ganglia injury manifests as headache, nausea, vomiting, limb numbness, dysarthria and visual impairment, with severity contingent upon hemorrhage volume and location (2). This life-threatening emergency causes significant neurological deficits. Surgical intervention remains the cornerstone of management, reducing mortality by rapidly alleviating cerebral compression and intracranial hypertension to salvage neurological function. Current guidelines for spontaneous intracerebral hemorrhage emphasize individualized neurosurgical decision-making, particularly in patients with large hematoma volume, neurological deterioration, intraventricular extension, or hydrocephalus requiring cerebrospinal fluid diversion (3).

Craniotomy for hematoma evacuation is the primary surgical intervention. Conventional transcortical approaches often necessitate traversing functional brain parenchyma, increasing iatrogenic injury risk (4, 5). In contrast, the trans-sylvian approach under microscopy, accessing the hematoma via the sylvian fissure and insular cortex, offers superior precision in accessing deep-seated hematomas, minimizing parenchymal injury, while facilitating more effective microsurgical hemostasis to substantially reduce rebleeding risk (6, 7). This concept is consistent with recent surgical evidence suggesting that the potential benefit of intracerebral hemorrhage evacuation depends not only on the extent of clot removal, but also on minimizing procedure-related injury through an appropriate surgical corridor (8, 9). However, when intraventricular rupture causes obstructive hydrocephalus, hematoma clearance alone often fails to address this complication. Uncontrolled hydrocephalus drives persistent intracranial hypertension, exacerbating neurological damage and severely compromising outcomes (10, 11). After intraventricular hemorrhage, ventricular blood clots, blood degradation products, ependymal injury, inflammatory activation, and impaired CSF absorption may jointly contribute to acute or delayed hydrocephalus (12–15). Thus, integrated strategies for simultaneous management are imperative.

Lamina terminalis (LT) ventriculostomy establishes direct communication between the third ventricle and subarachnoid space via fenestration at the thinnest natural barrier (16). Previous studies of lamina terminalis fenestration, mainly in aneurysmal subarachnoid hemorrhage, suggest that this procedure may facilitate CSF circulation and clot clearance through an anterior ventriculostomy, although its ability to prevent long-term shunt-dependent hydrocephalus remains controversial (17, 18).

This study aims to determine whether a combined approach, trans-sylvian hematoma evacuation plus LT ventriculostomy (combined group), offers superior outcomes versus isolated trans-sylvian evacuation (control group). Propensity score matching (PSM) adjusted for baseline confounders to objectively evaluate therapeutic efficacy, providing evidence-based guidance for clinical practice.

## Methods

### Study aim, design, and setting

This retrospective cohort study evaluated the clinical efficacy of a combined surgical approach for hypertensive basal ganglia hemorrhage (HBGH) with intraventricular extension at Xiangdong Hospital Affiliated to Hunan Normal University between October 2020 and February 2024. Patients were categorized into those who underwent isolated trans-sylvian hematoma evacuation (October 2020–October 2021; *n* = 35) as the control group and those who received trans-sylvian evacuation combined with lamina terminalis ventriculostomy (LTV) (January 2023–February 2024; *n* = 35) as the combined group. The final analysis was retrospective in nature, although the two cohorts were derived from different treatment periods. All included patients met the AHA/ASA diagnostic criteria for hypertensive intracerebral hemorrhage, had preoperative CT confirming basal ganglia hematoma ≥30 mL with intraventricular extension, required surgical intervention within 24 h of symptom onset, and had postoperative imaging available for assessment of hematoma evacuation and postoperative rebleeding. Exclusion criteria included non-hypertensive etiologies of hemorrhage (traumatic, neoplastic, or vascular malformation–related), major cardiac, hepatic, or renal dysfunction precluding surgery, concurrent cerebral infarction or intracranial aneurysm, and postoperative death within 24 h. The study protocol was approved by the Institutional Ethics Committee (Approval No. 2022-401), and informed consent was waived due to the retrospective design and anonymized data collection.

### Surgical procedures

#### Control group

Microscopic trans-sylvian hematoma evacuation via pterional craniotomy. The sylvian fissure was dissected to access the hematoma through the insular cortex, followed by meticulous hemostasis and placement of a 14Fr drainage tube within the basal ganglia hematoma cavity. Bone flap was removed if brain herniation exceeded >1 cm beyond the bone edge; otherwise, it was repositioned. Dural closure under reduced tension.

#### Combined group

**Figure 1:**
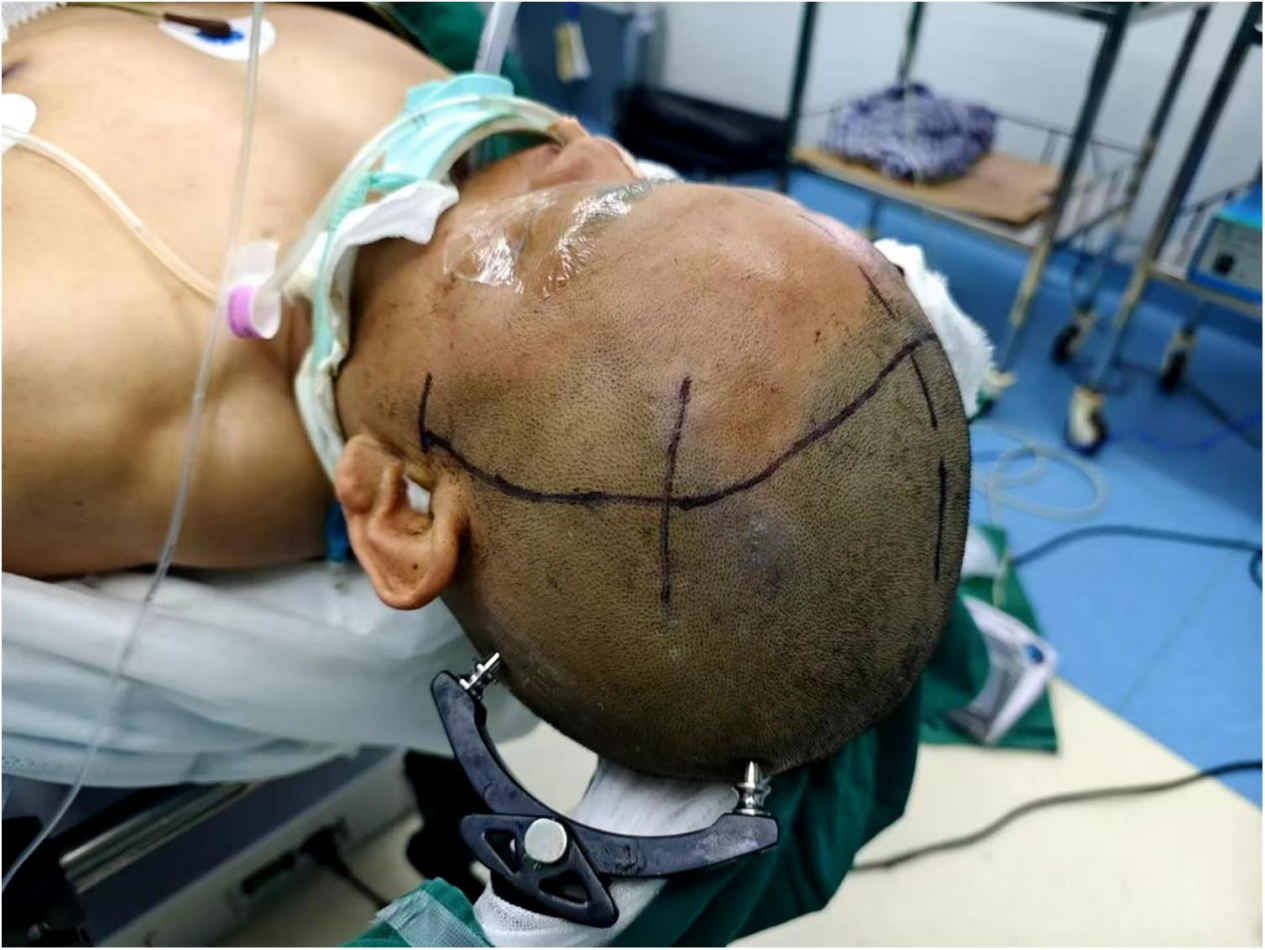
Intraoperative photograph demonstrating patient positioning, trans-sylvian operative planning, exposure of the lamina terminalis, and creation of the lamina terminalis ventriculostomy during trans-sylvian hematoma evacuation.

Following hematoma evacuation via the identical trans-sylvian approach as the control group, the carotid cistern, optic nerve, optic chiasm, and anterior communicating region were carefully exposed under microscopic visualization. A self-retaining retractor was used only when necessary to obtain adequate exposure of the lamina terminalis, which was identified as a thin, slightly bulging pale-blue membrane posterior to the optic chiasm. After confirming the midline anatomical orientation, a 5–6 mm fenestration was created sharply in the lamina terminalis. Continuous cerebrospinal fluid outflow from the third ventricle confirmed successful ventriculostomy (Figure 1). The indication for LTV was not based solely on the amount of visible intraventricular blood on a single postoperative CT image; rather, LTV was considered in patients with basal ganglia hematoma and ventricular extension in whom impaired CSF circulation, ventricular obstruction, or subsequent hydrocephalus risk was clinically relevant. A 14-Fr subdural drainage tube was then placed in the sylvian fissure cistern. Bone flap management adhered to the identical criteria as the control group. Dural closure was subsequently performed under reduced tension.

#### Postoperative management (both groups)

**Figure 2:**
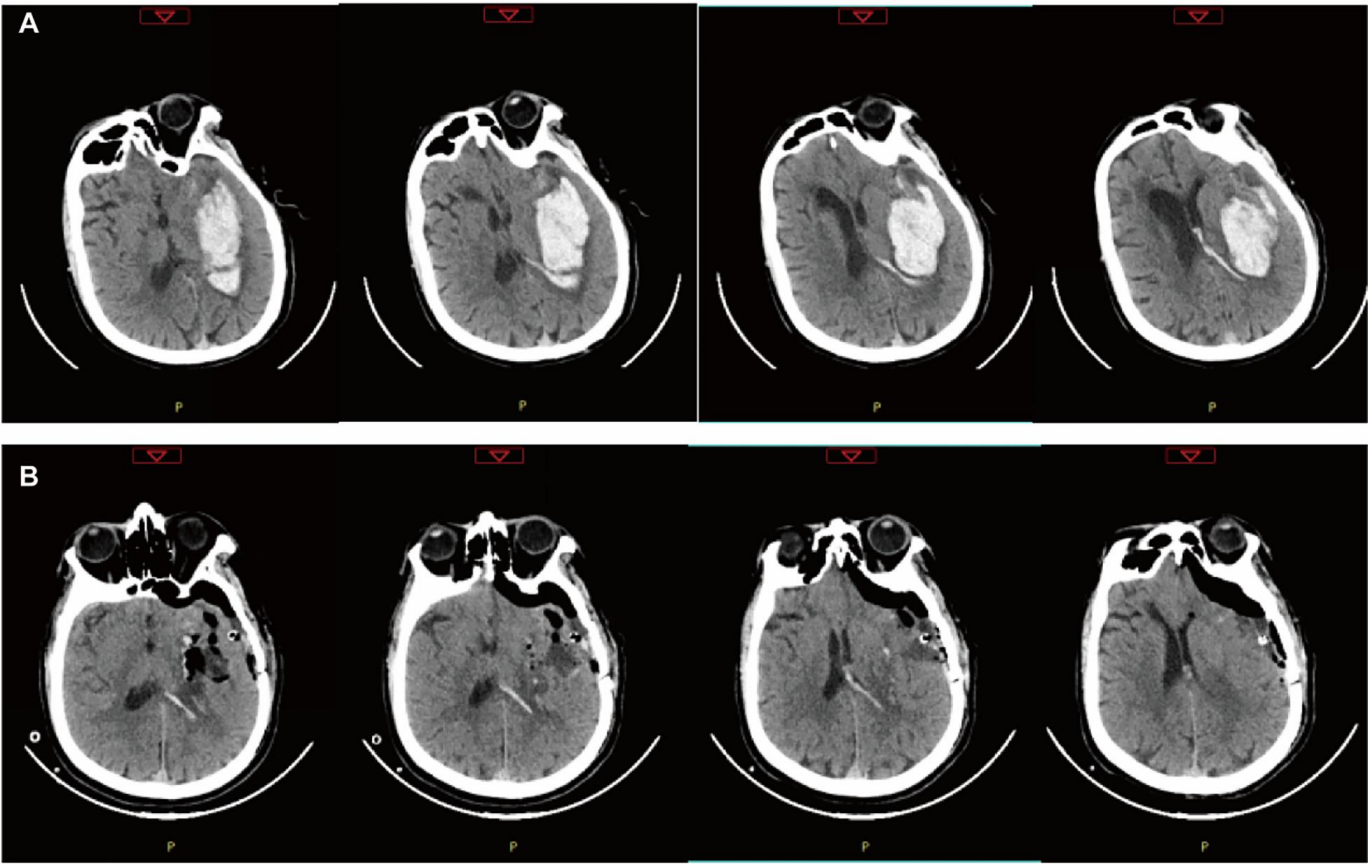
Representative preoperative **(A)** and postoperative **(B)** CT images demonstrating hematoma evacuation after combined surgical intervention. The figure illustrates surgical decompression and does not represent a formal assessment of hydrocephalus severity or the indication threshold for LTV.

All patients received standardized postoperative care: intravenous mannitol (125 mL every 6 h) combined with glycerol fructose (250 mL every 12 h) for intracranial pressure control; prophylactic cefazolin (2 g IV every 8 h) for 72 h to prevent infection; enteral nutrition initiated within 24 h with vitamin B12 supplementation; pantoprazole (40 mg IV daily) for stress ulcer prophylaxis; strict maintenance of systolic blood pressure <140 mmHg; CT scans at 12 h postoperatively (Figure 2) and hourly neurological assessments for 72 h.

### Data collection

#### Baseline data

All demographic and clinical data were collected from electronic medical records. Demographic data included age, sex and body mass index (BMI, categorized per WHO criteria: <18.5 underweight, 18.5–24.9 normal, ≥25 overweight/obese). Comorbidities encompassed hypertension (systolic BP ≥140 mmHg or antihypertensive use), diabetes mellitus (fasting glucose ≥7.0 mmol/L or glucose-lowering agents), and smoking status (current/former/never).

#### Surgical and hospitalization data

Intraoperative data were extracted from anesthesia and surgical records, including initial hematoma volume (mL, calculated using the ABC/2 method on preoperative CT scans), operative time (minutes, from skin incision to closure), intraoperative blood loss (mL, quantified via suction canister and swab weighing) and length of stay in hospital (days).

#### Hemodynamic parameters

Middle cerebral artery hemodynamics were assessed via transcranial Doppler (TCD) preoperatively and on postoperative days 7 and 14. Parameters recorded were mean flow velocity (Vm, cm/s) and pulsatility index (PI), with measurements performed by certified technicians using a 2-MHz Doppler probe.

#### Laboratory markers

Blood tests on postoperative day 1 included neutrophil-to-lymphocyte ratio (NLR) and lymphocyte count (LYM,  × 10⁹/L). Inflammatory biomarkers included c-reactive protein (CRP, mg/L), interleukin-6 (IL-6, pg/mL) and interleukin-10 (IL-10, ng/mL) were measured using standardized immunoassays.

#### Therapeutic outcomes

Therapeutic outcomes were evaluated using three components: functional recovery, quality-of-life assessment, and postoperative complications. Functional recovery was assessed using the Activities of Daily Living (ADL) grading system, categorized into five levels: I, full recovery; II, mild disability; III, mobility requiring assistance; IV, conscious but bedridden; and V, vegetative state or death. The effective rate was calculated as ADL grades I–II divided by the total number of cases. Quality of life was measured using the EORTC QLQ-C30 (Version 3.0), including physical, role, emotional, cognitive, and social functioning and symptom-related domains. Raw scores were standardized to a 0–100 scale according to the scoring method used in the institutional dataset, and assessments were conducted at discharge and at 1, 6, and 12 months postoperatively by trained interviewers. Because EORTC QLQ-C30 was originally developed for oncology populations rather than intracerebral hemorrhage, the results were interpreted as exploratory quality-of-life data. Complications during hospitalization and follow-up were systematically recorded, including pulmonary infection, gastrointestinal bleeding, epilepsy, brain herniation, postoperative rebleeding, and other clinically confirmed events. Pulmonary infection was diagnosed according to new pulmonary infiltrates on chest imaging together with compatible clinical manifestations and laboratory findings. Gastrointestinal bleeding was defined as clinically evident upper or lower gastrointestinal hemorrhage requiring medical treatment. Postoperative rebleeding was confirmed by repeat cranial CT.

### Statistical analysis

Statistical analyses were performed using SPSS 27.0 (IBM Corp., Armonk, NY, USA). Continuous variables were tested for normality using the Shapiro–Wilk test. Normally distributed variables are presented as mean ± standard deviation (SD) and compared using independent-sample t-tests. Non-normally distributed variables are presented as median (interquartile range, IQR) and compared using Mann–Whitney U tests. Categorical variables are summarized as frequency (percentage) and compared using *χ*^2^ tests (expected cell counts >5) or Fisher's exact tests (expected cell counts ≤5). Propensity score matching (PSM) was conducted using a 1:1.5 nearest-neighbor algorithm with a caliper width of 0.2 to reduce baseline imbalance while preserving statistical power in the context of a limited sample size. The propensity score model included variables available in the institutional database, including age, sex, body mass index, hypertension history, diabetes history, smoking status, initial hematoma volume, TNF-α, IL-6, IL-10, lymphocyte count, neutrophil-to-lymphocyte ratio, and C-reactive protein. Post-matching balance was assessed using standardized mean differences (SMD), with SMD <0.1 considered acceptable for the available covariates. Important prognostic variables, including preoperative Glasgow Coma Scale score, ICH score, Graeb score, intraventricular hemorrhage burden, and hydrocephalus severity, were not consistently available for all patients and therefore could not be incorporated into the matching model. Consequently, residual confounding cannot be completely excluded (19, 20).

## Results

### Intergroup baseline comparison and PSM adjustment

Before PSM, significant differences were observed in age, hypertension history, smoking status, and IL-10 levels between the two groups (*P* < 0.05), whereas sex, BMI, diabetes history, initial hematoma volume, TNF-α, IL-6, lymphocyte count, NLR, and CRP were comparable (*P* > 0.05). Because of these baseline imbalances and the limited sample size, propensity score matching was performed using a 1:1.5 nearest-neighbor algorithm (caliper=0.2). A total of 70 eligible patients were screened and included in the original cohort. After matching, 20 control patients and 30 combined-treatment patients remained for analysis. For the available covariates, standardized mean differences after matching were <0.1, and no statistically significant differences remained across the recorded demographic and clinical variables (all *P* > 0.05). However, unrecorded severity-related variables could not be balanced and were considered in the interpretation of the results. Details are shown in Table 1.

**Table 1:** Comparison of general data between the two patient groups (mean ± SD).

Indicator		Before PSM				After PSM			
		Control group (*n* = 35)	Combined group (*n* = 35)	*t/χ* ^2^	*P* value	control group (*n* = 20)	Combined group (*n* = 30)	*t/χ* ^2^	*P* value
Age (years)		58.48 ± 10.55	51.12 ± 9.54	3.061	0.003	58.16 ± 10.13	57.94 ± 9.46	0.078	0.938
Sex	Male	20	14	2.059	0.151	10	12	0.487	0.784
	Female	15	21			10	18		
BMI (kg/m^2^)		21.57 ± 2.45	21.34 ± 2.22	0.412	0.682	21.46 ± 2.45	21.67 ± 2.31	0.307	0.760
History of hypertension	Yes	6	15	5.510	0.019	5	8	0.017	0.991
	N/A	29	20			15	22		
History of diabetes	Yes	5	10	2.121	0.145	3	5	0.024	0.987
	N/A	30	25			17	25		
Smoking	Yes	15	5	5.202	0.023	8	10	0.232	0.891
	N/A	20	25			12	20		
Initial hematoma volume (ml)		40.58 ± 10.22	40.39 ± 9.29	0.081	0.935	40.08 ± 10.27	40.29 ± 8.77	0.077	0.939
TNF-α(ng/ml)		2.30 ± 0.28	2.35 ± 0.37	0.638	0.526	2.29 ± 0.52	2.33 ± 0.41	0.303	0.763
IL-6(pg/ml)		76.54 ± 5.27	76.08 ± 5.22	0.367	0.715	76.45 ± 2.43	76.05 ± 2.17	0.609	0.546
IL-10(ng/ml)		42.22 ± 1.28	42.99 ± 1.31	2.487	0.015	42.43 ± 1.15	42.37 ± 1.32	0.166	0.869
LYM( × 10^9^/L)		0.92 ± 0.22	1.04 ± 0.54	1.218	0.228	0.91 ± 0.34	1.03 ± 0.41	1.083	0.284
NLR		4.15 ± 0.55	4.29 ± 0.65	0.973	0.334	4.16 ± 0.72	4.27 ± 0.97	0.433	0.667
CRP (mg/L)		4.77 ± 0.72	4.92 ± 0.57	0.966	0.337	4.56 ± 0.76	4.82 ± 0.74	1.204	0.234

The combined group received trans-sylvian hematoma evacuation with lamina terminalis ventriculostomy (LTV), and the control group underwent isolated trans-sylvian hematoma evacuation.

### Cerebral hemodynamic outcomes

Before surgery, mean flow velocity (Vm) and pulsatility index (PI) of the middle cerebral artery did not differ significantly between the two groups (*P* > 0.05). At postoperative day 7, the combined group showed significantly higher Vm (75.51 ± 1.46 cm/s vs. 65.37 ± 2.71 cm/s; *P* < 0.001) and lower PI (0.87 ± 0.06 vs. 0.94 ± 0.03; *P* < 0.001) compared with the control group. By day 14, these differences further widened, with Vm (79.63 ± 2.34 cm/s vs. 69.46 ± 2.92 cm/s; *P* < 0.001) markedly elevated and PI (0.71 ± 0.07 vs. 0.88 ± 0.05; *P* < 0.001) significantly reduced in the combined group. These findings indicate that the combined surgical approach resulted in greater postoperative improvement in cerebral blood flow and reduced vascular resistance (Table 2).

**Table 2:** Comparison of middle cerebral artery blood flow parameters before operation, and 7 and 14 days after operation (mean ± SD).

Group	Flow velocity (Vm) (cm/s)			Pulsatility index (PI)		
	Pre-operation	Post-operation (days)		Pre-operation	Post-operation (days)	
		7	14		7	14
control group	38.13 ± 2.02	65.37 ± 2.71	69.46 ± 2.92	1.53 ± 0.11	0.94 ± 0.03	0.88 ± 0.05
combined group	37.27 ± 2.10	75.51 ± 1.46	79.63 ± 2.34	1.49 ± 0.12	0.87 ± 0.06	0.71 ± 0.07
*t* value	1.440	17.150	13.628	1.790	4.820	9.370
*P* value	0.156	0.000	0.000	0.080	0.000	<0.001

### Therapeutic efficacy

Therapeutic efficacy differed significantly between the two groups. In the control group, ADL grades I–II were achieved in 7/20 patients (35.00%), whereas poorer outcomes remained relatively common. In contrast, the combined group demonstrated better ADL-based functional recovery, with 23/30 patients (76.67%) achieving ADL grades I–II and fewer patients in grades IV–V. The overall distribution of therapeutic efficacy scores showed a statistically significant improvement in the combined group compared with the control group (*χ*^2^ = 8.681, *P* = 0.003) (Table 3).

**Table 3:** Comparison of therapeutic efficacy between the two patient groups.

Group	Score					Therapeutic efficacy (%)
	Ⅰ	Ⅱ	Ⅲ	Ⅳ	Ⅴ	
control group	5	2	6	4	3	35.00
combined group	12	11	3	2	2	76.67
*Z*/*χ*^2^ value	8.681					
*P* value	0.003					

I: complete recovery of daily living abilities; II: minor disabilities that do not affect independent living, can manage normal life with some assistance; III: disabilities requiring external support for mobility; IV: patient is conscious but bedridden; V: vegetative state or death. Effective rate = (I + II)/total number of cases × 100.00%.

### Complication profiles

The overall complication rate was significantly lower in the combined group than in the control group (6.67% vs. 55.00%, *χ*^2^ = 14.571, *P* < 0.001). In the control group, gastrointestinal bleeding occurred in 1/20 patients (5.00%), pulmonary infection in 5/20 (25.00%), epilepsy in 2/20 (10.00%), and postoperative rebleeding in 3/20 (15.00%). In contrast, the combined group reported only two cases of postoperative rebleeding (2/30, 6.67%) and no gastrointestinal bleeding, pulmonary infection, epilepsy, or brain herniation. These findings indicate a significantly lower recorded postoperative complication burden in patients receiving the combined surgical approach. However, detailed information regarding ICU length of stay, external ventricular drainage duration, and subsequent CSF diversion procedures was not consistently available in the retrospective dataset (Table 4).

**Table 4:** Comparison of complication rates between the two patient groups.

Complications	Control group (%)	Combined group (%)	*χ*^2^ value	*P* value
Gastrointestinal bleeding	5.00	0	–	–
Pulmonary infection	25.00	0	–	–
Epilepsy	10.00	0	–	–
Brain herniation	0	0	–	–
Postoperative rebleeding	15.00	6.67	–	–
Incidence rate	55.00	6.67	14.571	<0.001

### Quality of life assessment

EORTC QLQ-C30 scores differed significantly between the two groups during postoperative follow-up. At discharge, the combined group had a mean score of 19.51 ± 7.46, compared with 25.49 ± 5.33 in the control group (t = 3.093, *P* = 0.003). Similar intergroup differences were observed at 1 month (29.77 ± 5.75 vs. 35.67 ± 5.21; t = 3.688, *P* = 0.001), 6 months (32.37 ± 5.38 vs. 49.27 ± 5.34; t = 10.914, *P* < 0.001), and 12 months (49.72 ± 5.34 vs. 53.46 ± 5.92; t = 2.323, *P* = 0.024) (Table 5). According to the scoring direction described in the Methods section, these results do not support a superior global quality-of-life outcome in the combined group despite the observed improvement in ADL-based functional recovery. Therefore, the quality-of-life findings should be interpreted cautiously and require confirmation using stroke-specific instruments such as EQ-5D or SF-36 in future studies.

**Table 5:** Comparison of EORTC QLQ-C30 global scores between the two patient groups (mean ± SD).

Group	At discharge	1 month after discharge	6 months after discharge	12 months after discharge
control group	25.49 ± 5.33	35.67 ± 5.21	49.27 ± 5.34	53.46 ± 5.92
combined group	19.51 ± 7.46	29.77 ± 5.75	32.37 ± 5.38	49.72 ± 5.34
*t* value	3.093	3.688	10.914	2.323
*P* value	0.003	0.001	0.000	0.024

## Discussion

In this study, trans-sylvian hematoma evacuation combined with lamina terminalis ventriculostomy (LTV) was associated with better postoperative outcomes than isolated trans-sylvian hematoma evacuation in selected patients with hypertensive basal ganglia hemorrhage (HBGH) and intraventricular extension. After propensity score matching, patients in the combined group showed more favorable cerebral hemodynamic parameters, better ADL-based functional recovery, and a lower overall postoperative complication rate. These findings suggest that simultaneous management of both the deep parenchymal hematoma and cerebrospinal fluid (CSF) circulation disturbance may have potential clinical value in this patient population. However, because this was a non-randomized single-center cohort study, these findings should be interpreted as preliminary and hypothesis-generating rather than confirmatory. The potential advantage of the combined procedure is supported by the pathophysiological characteristics of HBGH with ventricular rupture. In such patients, neurological deterioration is not caused solely by the hematoma itself, but also by intraventricular blood, impaired CSF circulation, and persistent intracranial hypertension. Once blood enters the ventricular system, clot obstruction may occur at the foramen of Monro, aqueduct, or fourth ventricular outlets. In addition, blood degradation products can induce ependymal injury, inflammatory responses, altered CSF secretion and absorption, and scarring of CSF pathways, all of which may aggravate hydrocephalus and secondary brain injury. Therefore, even after adequate clot evacuation, postoperative recovery may remain limited if ventricular obstruction and CSF flow disturbance are not effectively addressed. In this context, LTV may help restore CSF circulation through a natural anatomical pathway and improve the intracranial environment after surgery. By fenestrating the lamina terminalis, LTV creates an anterior communication between the third ventricle and basal cisterns, which may facilitate CSF redistribution and reduce ventricular pressure. It should be emphasized that the indication for LTV in the present study was not based solely on the amount of intraventricular blood visible on one representative CT image. Instead, LTV was considered in patients with HBGH and ventricular extension in whom CSF circulation disturbance or hydrocephalus risk was considered clinically relevant. However, because most available evidence for lamina terminalis fenestration comes from aneurysmal subarachnoid hemorrhage rather than hypertensive basal ganglia hemorrhage, the present findings should be regarded as preliminary and disease-specific validation remains necessary. This may partly explain why the combined group showed higher postoperative mean flow velocity and lower pulsatility index, indicating improved cerebral perfusion and reduced vascular resistance.

The present findings also differ from studies focusing on ventricular drainage, intraventricular thrombolysis, or hematoma evacuation alone. In CLEAR III and other major clinical studies of intracerebral or intraventricular hemorrhage, improvement in functional outcome was not uniform across all treated populations, although clot clearance, mortality-related signals, and patient-selection issues remained clinically relevant (21–23). This suggests that CSF diversion, ventricular clot management, or hematoma evacuation alone may be insufficient for functional recovery when both deep parenchymal hematoma mass effect and ventricular CSF circulation disturbance coexist. In contrast, the present combined procedure attempts to address both components simultaneously: basal ganglia hematoma compression and ventricular CSF circulation disturbance. Nevertheless, direct comparison with these trials should be made cautiously because of differences in study design, patient selection, treatment strategy, and outcome assessment.

A further important finding of the present study was the lower complication burden in the combined group. This result is clinically important because EVD-related management of IVH may be associated with catheter occlusion, infection, repeated manipulation, and prolonged intensive care exposure (24). The one-stage trans-sylvian evacuation plus LTV strategy may reduce the need for delayed or repeated CSF diversion procedures, although this hypothesis requires confirmation using standardized data on EVD use, catheter duration, intracranial pressure monitoring, and infection surveillance (25). Postoperative complications are major determinants of poor outcome after intracerebral hemorrhage, particularly in patients with severe neurological injury. In our cohort, the combined group had a markedly lower overall complication rate, with fewer cases of pulmonary infection, epilepsy, and postoperative rebleeding. Although the mechanisms underlying these reductions cannot be fully clarified in a retrospective analysis, they may be related to more effective intracranial pressure control, reduced need for secondary intervention, and earlier stabilization of neurological status. Clinically, this is meaningful because prevention of early postoperative complications may facilitate rehabilitation and improve overall recovery. However, because detailed information regarding ICU length of stay, time-at-risk, EVD duration, and catheter-related events was not consistently available, the complication findings should be interpreted cautiously.

The trans-sylvian approach itself may also contribute to the feasibility and value of this combined strategy. Compared with conventional transcortical routes, the trans-sylvian corridor utilizes a natural anatomical space and may reduce additional injury to functional brain tissue. More importantly, after hematoma evacuation, the lamina terminalis can be exposed within the same operative field under microscopic vision, allowing LTV to be performed without establishing a separate surgical pathway. This technical feature makes the combined procedure practical and may reduce the disadvantages associated with staged management of hematoma evacuation and CSF diversion. From a surgical perspective, such a one-stage strategy may be particularly useful in patients with ventricular extension, in whom delayed treatment of CSF circulation disturbance could adversely affect postoperative recovery. At the same time, the postoperative CT images in this study should be interpreted mainly as representative images showing hematoma evacuation and decompression, rather than as objective evidence of hydrocephalus severity or as the sole basis for performing LTV.

The better functional outcomes observed in the combined group further support the potential clinical relevance of this approach. Recovery after HBGH depends not only on removal of the hematoma, but also on limitation of secondary brain injury caused by sustained intracranial hypertension, impaired perfusion, ventricular CSF disturbance, and medical complications during the perioperative period. By improving postoperative intracranial conditions at an early stage, the combined procedure may create a more favorable basis for neurological recovery. Accordingly, the observed improvement in ADL-based outcomes is likely to reflect not only successful decompression, but also better postoperative physiological stabilization. However, the quality-of-life findings should be interpreted separately from the ADL results. In the present study, EORTC QLQ-C30 scores did not demonstrate a consistent advantage for the combined group, despite better ADL-based recovery. Therefore, the present data support improved functional recovery but do not prove superior global quality of life. In addition, EORTC QLQ-C30 was originally developed for oncology populations and may not be the most appropriate instrument for evaluating long-term outcomes after intracerebral hemorrhage. Future studies should use stroke-specific or general health-related quality-of-life instruments to confirm these findings.

Several limitations should be acknowledged. First, this was a single-center study with a relatively small sample size, which may limit the robustness and generalizability of the findings. Second, although propensity score matching was performed to reduce baseline imbalance, residual confounding from unmeasured variables cannot be excluded. In particular, important prognostic variables such as preoperative Glasgow Coma Scale score, ICH score, intraventricular hemorrhage burden, Graeb score, and hydrocephalus severity were not consistently available and therefore could not be incorporated into the matching model. Third, the two groups were enrolled during different time periods, and temporal differences in perioperative management, surgical experience, or postoperative care may have influenced the observed outcomes. Fourth, data regarding shunt dependency, delayed hydrocephalus, VP shunt placement, EVD duration, and subsequent CSF diversion procedures were not systematically collected. Therefore, this study cannot determine whether LTV reduces long-term shunt-dependent hydrocephalus. Finally, longer-term follow-up with standardized neurological and imaging assessment is still needed to determine whether the combined procedure can improve long-term neurological prognosis. Therefore, larger prospective multicenter studies are warranted to further validate the efficacy, indications, and long-term benefit of this combined surgical strategy.

## Conclusion

In conclusion, trans-sylvian hematoma evacuation combined with LTV appears to be a feasible surgical option for selected patients with HBGH and intraventricular extension. By addressing both hematoma removal and CSF circulation disturbance within a single operative corridor, this approach was associated with improved cerebral hemodynamics, better ADL-based recovery, and fewer recorded postoperative complications. Because of the retrospective design, non-contemporaneous cohorts, and unavailable severity and shunt-dependency data, these findings should be regarded as preliminary and require confirmation in prospective multicenter studies.

## Data Availability

The original contributions presented in the study are included in the article/Supplementary Material, further inquiries can be directed to the corresponding author.
